# Supplementary material for: Complexome profiling on the Chlamydomonas lpa2 mutant reveals insights into PSII biogenesis and new PSII associated proteins
Source: J Exp Bot. 2021 Aug 26;73(1):245–62. doi: 10.1093/jxb/erab390 (PMC8730698; doi:10.1093/jxb/erab390)
Supplement: erab390_suppl_Supplementary_Dataset_S1 [file erab390_suppl_supplementary_dataset_s1.zip › Supplemental Dataset 1 - Excel List and all profiles/plots/RPL10A_Cre02.g101350.html]

Trivial name: RPL10A  
  
Euclidean distance: 9293.71  
Mean Intensity (WT): 811.77  
Mean Intensity (Mut): 926.66  
Distance: 10.03  
  
MapMan: protein.synthesis.ribosomal protein.eukaryotic.60S subunit.L10A;protein.synthesis.ribosomal protein.prokaryotic.unknown organellar.50S subunit.L10A;protein.synthesis.ribosomal protein.prokaryotic.unknown organellar.50S subunit.L10A  
  
p value of intensity sums Welch test: 0.8379
